# Synergistic Regulation of Postharvest Physiology and Biochemical Quality of Peach (*Prunus persica* L.) Through Salicylic Acid and Plant‐Based Edible Treatments

**DOI:** 10.1002/pei3.70180

**Published:** 2026-06-25

**Authors:** Sima Shajari, Hamid Madani, Leila Pourhoseini, Farzad Paknejad, Mohammad Nabi Ilkaee

**Affiliations:** ^1^ Department of Horticulture Science Ka.C., Islamic Azad University Karaj Iran; ^2^ Department of Agronomy Ar.C., Islamic Azad University Arak Iran; ^3^ Department of Agronomy and Plant Breeding Ka.C., Islamic Azad University Karaj Iran

**Keywords:** natural bioactive compounds, natural preservation strategies, phenolic metabolism, storage stability

## Abstract

Postharvest deterioration represents a major challenge in maintaining the market quality of peach fruit, and the application of environmentally benign treatments such as edible coatings has gained increasing attention as an effective strategy to extend storage life. Accordingly, the present study evaluated the effects of several natural coating materials on the postharvest quality of peach ( *Prunus persica* L.). The experimental treatments consisted of salicylic acid at three concentrations (0, 1, and 2 mM), *Aloe vera* gel at 0%, 20%, and 30%, and clove extract at 0, 150, and 300 ppm. Overall, the application of edible coatings significantly enhanced fruit storability and quality attributes compared with untreated control fruit. Among the treatments, fruit treated with 2 mM salicylic acid and 300 ppm clove extract exhibited the highest fruit weight (89.66 g). Whereas the fruits without salicylic acid application showed the greatest fruit weight loss (15.55%) and the highest vitamin C (32.02 mg 100 g^−1^ FW) was obtained at 2 mM salicylic acid treatment. In terms of clove extract treatments, fruit coated with 300 ppm clove extract maintained the greatest fruit texture firmness (3.69 kg cm^−2^), total phenolic content (219.88 mg GAE 100 g^−1^ FW), vitamin C (mg 100 g^−1^ FW), and total antioxidant capacity (446.94 μmol TE g^−1^ FW), whereas no application treatment recorded the highest fruit weight loss (14.72%) and total acidity (4.16 g malic acid 100 g^−1^ FW). In conclusion, the findings demonstrate that natural edible coatings, particularly when applied in combination, are effective in reducing oxidative stress, suppressing enzymatic browning, and improving postharvest quality, thereby representing a promising and sustainable approach for extending the shelf life of peach fruit. These findings suggest that activation of antioxidant system likely stimulated by salicylic acid and supplemented by *aloe vera* or clove extract was a central mechanism underlying quality preservation.

## 1 Introduction

Peach ( *Prunus persica* L.) is one of the most widely cultivated and consumed temperate fruit crops worldwide, highly valued for its distinctive aroma, sweet taste, and nutritional composition. In addition to primary metabolites such as carbohydrates and organic acids, peaches are a significant source of health‐promoting phytochemicals, including carotenoids, phenolic compounds, and flavonoids, which contribute to antioxidant capacity and human health benefits. However, this fruit's high metabolic activity after harvest renders it susceptible to rapid quality deterioration, leading to shortened shelf life and increased postharvest losses in supply chains across the globe (Aaqil et al. 2024).

Postharvest deterioration in peach fruit begins soon after harvest due to ongoing respiration, ethylene production, and cell wall degradation that continue even after detachment from the parent plant (Taher et al. 2022). These physiological processes result in moisture loss, textural softening, breakdown of cell wall polysaccharides, surface wrinkling, and internal browning, significantly diminishing fruit marketability and consumer acceptance. In addition, the thin epidermis of peaches makes them vulnerable to mechanical injuries and microbial invasion during handling, transport, and storage. The combined effects of metabolic deterioration and pathogenic attack cause substantial quantitative and qualitative losses, which can reach up to 40%–50% in regions lacking adequate postharvest handling infrastructure (Ezeike et al. 2022).

Traditional methods aimed at retarding fruit senescence and decay primarily rely on low‐temperature storage and modified atmosphere conditions. While refrigeration can reduce respiration rates and slow biochemical changes, peaches are also sensitive to chilling injury at low temperatures, which can disrupt cell membrane integrity, delay ripening unevenly, and cause off‐flavors and textural disorders upon transfer to ambient conditions (Aaqil et al. 2025). These limitations highlight the need for additional postharvest technologies that not only slow physiological deterioration but also preserve the sensory and nutritional quality of peaches during extended storage and distribution.

In recent years, edible coatings have emerged as a promising postharvest technology to enhance fruit quality and extend shelf life in an eco‐friendly manner. Edible coatings are thin, consumable layers of biopolymers applied to the surface of fruits to form a semi‐permeable barrier. These barriers can reduce moisture loss, decrease oxygen influx, and slow down ethylene diffusion, collectively retarding the ripening process and reducing oxidative damage. Additionally, edible coatings may incorporate natural bioactive compounds with antioxidant and antimicrobial properties, further enhancing their protective effects during storage (Hasan et al. 2021).

Naturally sourced materials such as polysaccharides, proteins, lipids, and composite matrices have been extensively evaluated for edible coating applications (Aaqil et al. 2024, 2025; Ezeike et al. 2022; Taher et al. 2022). Within this group, polysaccharide coatings—especially those prepared from *Aloe vera* (AV) gel—have emerged as promising candidates because of their structural integrity, eco‐friendly nature, and inherent biological activity. AV gel is composed mainly of water along with a complex mixture of polysaccharides, vitamins, minerals, and phenolic compounds that can contribute to moisture retention, antioxidant activity, and microbial inhibition when applied as a coating. Research has shown that AV‐based coatings can effectively delay weight loss, preserve firmness, and reduce respiration rate in various fruits under storage conditions (Hasan et al. 2021). The effectiveness of AV coatings in preserving the postharvest quality of climacteric fruit has been demonstrated in studies on various horticultural crops. For example, composite edible films containing AV have been found to reduce weight loss and maintain quality attributes in strawberries and figs during cold storage by modulating gas exchange and reinforcing antioxidant defenses (Tabing et al. 2024). These natural coatings not only help maintain physicochemical characteristics such as total soluble solids and titratable acidity but also improve nutritional quality by preserving phenolic and flavonoid contents, which are susceptible to oxidative degradation postharvest.

Beyond physical barriers, the integration of bioactive compounds such as plant hormones and signaling molecules into edible coatings has attracted considerable research attention. Salicylic acid (SA) is one such naturally occurring phenolic compound ubiquitous in plants, functioning as a key regulator of defense responses and stress tolerance. SA possesses antioxidant properties and modulates the synthesis of defense‐related enzymes, enhances the activity of phenylpropanoid pathways, and has been implicated in slowing ethylene biosynthesis (Jahani et al. 2021). Through these mechanisms, SA can delay fruit ripening, reduce membrane deterioration, and lessen susceptibility to postharvest pathogens (Keshavarz et al. 2016; Yousefzadeh et al. 2023). Recent studies have explored the use of SA both as an independent postharvest treatment and as part of composite edible coatings. For instance, edible coatings composed of gum arabic and polyvinylpyrrolidone incorporating SA significantly reduced the activities of cell wall‐degrading enzymes such as cellulase and pectinase in peach fruit, thereby mitigating tissue breakdown and delaying internal browning during shelf life at ambient temperature (Taher et al. 2022; Sabourifard et al. 2023). This approach also maintained structural integrity by limiting polyphenol oxidase activity, which is closely associated with enzymatic browning reactions. The combination of AV‐based coatings with SA or other bioactive treatments represents a synergistic strategy that leverages both barrier properties and biochemical modulation to improve postharvest outcomes. Studies on other fruit systems, including mango and guava, have demonstrated that integrating SA within an AV coating can preserve higher levels of total phenolic and antioxidant capacity, reduce respiration and ethylene production, and maintain firmness and visual quality compared with untreated controls (Morsalpour and Rastegar 2025), suggesting that composite treatments may be more effective than individual applications in enhancing storability and preserving nutritional and sensory quality.

Despite substantial progress in the development of SA, AV gel and clove extract ( *Syzygium aromaticum* ) (CE) applications (as a hormonal regulation, physical barrier formation and biochemical protection, respectively) in fruit preservation, research specifically focused on peach remains comparatively limited. Given the unique physiological characteristics of peach and its susceptibility to chilling injury and rapid softening, targeted investigation of combined postharvest treatments is crucial. Moreover, understanding how these treatments interact with intrinsic fruit metabolism, defense pathways, and antioxidant systems can inform optimized protocols for maintaining quality and extending shelf life (Rah‐Khosravani et al. 2017).

Accordingly, this study investigates the effects of natural edible coating treatments, applied both individually and in combination, on the postharvest quality characteristics of peach fruit. Through comparative evaluation of these treatments under controlled storage conditions, the study seeks to generate insight into sustainable postharvest approaches that extend shelf life, minimize quality deterioration, and preserve the nutritional and sensory attributes of peaches during storage.

## 2 Materials and Methods

### 2.1 Experimental Design and Treatments

This study was conducted as a factorial experiment based on a completely randomized design (CRD) with four replications on peach ( *Prunus persica* L. cv. Zafarani). The experimental treatments consisted of three factors. The first factor was SA at three levels: no application, 1, and 2 mM. The second factor was AV gel at three levels: no application, 20%, and 30%. The third factor was CE applied at three levels: no application, 150, and 300 ppm. Peach fruits of the “Zafarani” cultivar were harvested at commercial maturity from an orchard located in the central province of Arak, Iran. Care was taken to select fruits that were uniform in size, healthy, and free from mechanical damage, wounds, or disease. AV leaves used for gel extraction were obtained from a commercial farm in Arak. To prepare the CE, dried clove samples were first ground into powder and then extracted. AV coatings at different concentrations (20% and 30% w/w) were prepared by diluting the gel with distilled water, following the method described by Boudreau and Beland (2006). Prior to treatments, fruits were washed with distilled water, surface‐disinfected using a sodium hypochlorite solution (100 mg L^−1^) for 2 min, rinsed with sterile distilled water, and air‐dried at room temperature. The fruits were immersed in the respective treatment solutions (SA, AV gel, and CE), labeled, and air‐dried at room temperature. Control fruits received no coating. Each experimental unit consisted of five fruits, and the storage period lasted 4 weeks (20°C–25°C, 60%–70% RH).

### 2.2 Data Collection

#### 2.2.1 Fruit Weight and Amount of Fruit Weight Loss

Individual fruit weight was recorded at the beginning of storage and at each sampling interval using a digital analytical balance with 0.01 g precision. Initial fruit weight was recorded immediately after treatment application. Weight loss was calculated as the percentage reduction in fruit mass relative to the initial weight using the following equation (1) (Nasrin et al. 2025; Moradi‐Ghahderijani et al. 2017).

(1) $$\text{Weight loss}\;\left(\%\right):\frac{W_{0}-W_{t}}{W_{0}}\times 100$$

where *W*_0_ is the initial fruit weight (g) and *W*_*t*_ is the fruit weight at the corresponding storage time. Mean values were calculated from at least three replicate fruits per treatment.

#### 2.2.2 Fruit Texture Firmness and Fruit Flesh Percentage

Fruit texture firmness was determined using a digital texture analyzer or penetrometer equipped with a cylindrical stainless‐steel probe (typically 8 mm diameter). Before measurement, a small portion of peel was carefully removed from two opposite sides of each fruit at the equatorial region. Firmness was measured by penetrating the probe into the flesh to a constant depth at a uniform speed. The results were expressed as hardness (kg cm^−2^) and values were calculated as the mean of measurements obtained from at least three fruits, with two readings per fruit (Morsalpour and Rastegar 2025).

#### 2.2.3 Fruit Decay

Fruit decay was assessed visually by examining each fruit for symptoms of fungal growth, tissue breakdown, or visible rot. Fruits showing any signs of decay were considered decayed and removed from the marketable count. Fruit decay percentage was calculated using the following equation (2):

(2) $$\text{Fruit decay}\;\left(\%\right):\frac{\text{Number of decayed fruits}}{\text{Total number of fruits}}\times 100$$

#### 2.2.4 Total Phenolic Content

The Folin–Ciocalteu method was employed to measure total phenolic content, with results calculated from a gallic acid calibration curve and expressed as mg GAE per 100 g fresh weight (Masjedi et al. 2025).

#### 2.2.5 Vitamin C Content

Vitamin C content was quantified using the 2,6‐dichlorophenolindophenol (DCPIP) titrimetric method (Cao et al. 2025). Fresh fruit juice was extracted and filtered. A known volume of juice was titrated against standardized DCPIP solution until a persistent light pink endpoint was observed. Ascorbic acid concentration was calculated based on the volume of titrant consumed and expressed as milligrams of ascorbic acid per 100 g fresh weight (mg 100 g^−1^ FW).

#### 2.2.6 Total Antioxidant Capacity

Total antioxidant capacity was assessed using the DPPH (2,2‐diphenyl‐1‐picrylhydrazyl) radical scavenging assay (Keshavarz and Khodabin 2019). Fruit extracts were prepared in methanol, and a defined volume was mixed with DPPH solution. The mixture was incubated in the dark at room temperature, and absorbance was recorded at 517 nm. Antioxidant capacity was expressed as Trolox equivalent antioxidant capacity (μmol TE g^−1^ FW), calculated from a Trolox standard curve.

#### 2.2.7 Total Acidity

Total titratable acidity was determined by titration following standard analytical procedures. Juice was extracted from fruit flesh using a laboratory blender and filtered through cheesecloth to remove solid residues. A known volume of juice (usually 10 mL) was diluted with distilled water and titrated against 0.1 N sodium hydroxide (NaOH) solution using phenolphthalein as an indicator until a persistent light pink endpoint was reached (pH≈8.1). Total acidity was calculated and expressed as grams of malic acid per 100 g of fresh weight (g malic acid 100 g^−1^ FW) using the following equation (3):

(3) $$\mathrm{TA}:\frac{V\times N\times E}{W}\times 100$$

where *V* is the volume of NaOH consumed (mL), *N* is the normality of NaOH, *E* is the equivalent weight of malic acid, and *W* is the sample weight (g).

#### 2.2.8 Juice pH

Juice pH was measured using a calibrated digital pH meter. Prior to measurement, the pH meter was standardized using buffer solutions at pH 4.0 and 7.0. Fresh juice was extracted from peach flesh, filtered, and analyzed at room temperature. The electrode was rinsed with distilled water between samples to avoid cross‐contamination. pH values were recorded directly, and results were expressed as mean values from at least three replicates per treatment.

#### 2.2.9 Ammonium (NH_4_ ^+^) Content

Ammonium content in fruit tissue was determined using the phenol‐hypochlorite method (Zhang et al. 2020). Fruit samples were homogenized in distilled water and filtered. An aliquot of the extract was reacted with phenol and alkaline hypochlorite reagents and incubated at 37°C for color development. Absorbance was measured at 625 nm using a spectrophotometer. Ammonium concentration was calculated using an ammonium chloride standard curve and expressed as milligrams of ammonium per kilogram fresh weight (mg NH _4_ ^+^ kg^−1^ FW).

#### 2.2.10 Toluene

Toluene content was determined using gas chromatography (GC) following the method of Zhao et al. (2021) with slight modifications. Fruit tissue was homogenized and extracted with n‐hexane. The mixture was vortexed and centrifuged. The supernatant was filtered through a 0.45 μm membrane filter, and 1 μL was injected into a gas chromatograph equipped with a flame ionization detector (FID) and a capillary column (e.g., HP‐5, 30 m × 0.25 mm × 0.25 μm). Nitrogen (or helium) was used as the carrier gas. Quantification was performed using an external standard calibration curve prepared with known concentrations of toluene, and results were expressed as micrograms of toluene per kilogram fresh weight (μg kg^−1^ FW).

### 2.3 Statistical Analysis

All measurements were performed in triplicate, and the data were subjected to analysis of variance (ANOVA) using statistical software (SAS release 9.0 2002), and mean comparisons were conducted using Least Significant Difference tests (LSD) at a significance level of *p* ≤ 0.05. Excel software was used to draw graphs.

## 3 Results

### 3.1 Fruit Weight

**FIGURE 1:**
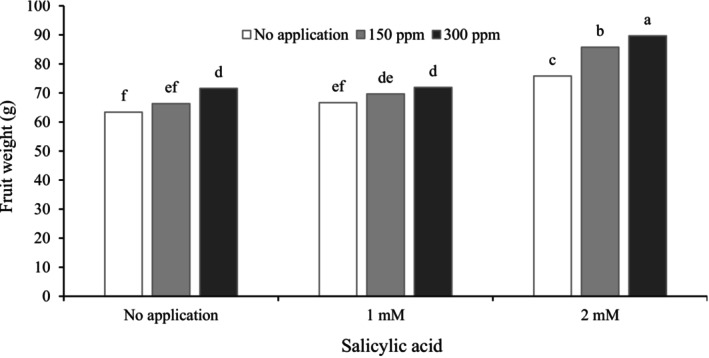
Mean comparison of interaction effect of salicylic acid × clove extract on fruit weight of peach ( *Prunus persica* L.). Means followed by the same letter are not significantly different by least significant difference test (*p* ≤ 0.05).

The interactions of SA × AV gel and SA × CE were significant for fruit weight (Table 1). Mean comparisons using the LSD test showed that the highest fruit weight was obtained in fruit treated with 2 mM SA combined with 30% AV gel, with an average value of 98.6 g (Table 2). Averaged across salicylic acid treatments, fruit weight was higher under 30% AV (86.0 g) than under 20% AV gel (74.59 g) and no AV application (67.37 g). At all AV gel levels, increasing SA concentration resulted in increased fruit weight. The interaction between SA and CE was also highly significant (*p* ≤ 0.01) for fruit weight (Table 1). Under SA treatments, application of CE improved fruit weight, placing fruit in the highest statistical group (Figure 1). The maximum fruit weight was observed in fruit treated with 2 mM SA and 300 ppm CE (89.66 g), representing increases of 19.79% and 20.26% compared with 1 mM SA × 300 ppm CE and no SA × 300 ppm CE treatments, respectively.

**TABLE 1:** Combined analysis of variance on some physiological traits of peach ( *Prunus persica* L.) as affected by salicylic acid, *aloe vera* gel, clove extract treatments.

Sources of variance	df	Fruit weight	Fruit weight loss	Fruit texture firmness	Fruit flesh	Decay	Total phenolic content	Vitamin C	Total antioxidant capacity	Total acidity	Juice pH	Ammonium content	Toluene
Block	2	97.39**	7.22ns	0.007ns	178**	3.03ns	481ns	37.46*	3654**	0.007ns	0.06ns	0.0008ns	0.011*
Salicylic acid (S)	2	2925**	542.81**	6.74**	2410**	565**	53377**	2640**	143342**	6.74**	1.11**	1.18**	0.83**
*Aloe vera* gel (A)	2	2282**	168.92**	1.52**	675**	383**	36402**	667**	36698**	1.52**	1.23**	0.09**	0.11**
Clove extract (D)	2	749**	20.34**	0.13**	122**	12ns	9237**	94.77**	3681**	0.13**	0.09ns	0.05**	0.05**
A × S	4	464**	2.75ns	0.11**	90**	33**	12720*	5.47ns	620ns	0.11**	0.20**	0.15**	0.16**
D × S	4	74.89**	3.46ns	0.008ns	31**	9.3ns	639.8ns	1.59ns	106ns	0.008ns	0.01ns	0.003ns	0.001ns
D × A	4	35.23ns	0.53ns	0.01ns	0.45ns	2.3ns	595ns	2.51ns	599ns	0.01ns	0.01ns	0.004ns	0.008ns
D × S × A	8	17.06ns	1.78ns	0.005ns	0.45ns	4.7ns	630ns	6.43ns	222ns	0.005ns	0.02ns	0.0009ns	0.0003ns
Error	78	20.6	3.49	0.006	4.3	8.94	308.09	9.52	257.2	0.017	0.03	0.007	0.003
Coefficient of variation (%)		6.19	15.19	2.21	2.7	10.16	8.57	13.02	3.86	3.38	4.70	22.8	16.37

*Note:* ns, ** and * and indicate no significance and significance at *p* levels of 0.01 and 0.05, respectively.

**TABLE 2:** Mean comparison of salicylic acid *aloe vera* gel treatments interaction on studied traits of peach ( *Prunus persica* L.).

Salicylic acid	*Aloe vera* gel	Fruit weight	Fruit texture firmness	Fruit flesh	Decay	Total phenolic content	Total acidity	Juice pH	Ammonium content	Toluene
		(g)	(kg cm^−2^)	(%)	(%)	(mg GAE 100 g^−1^ FW)	(g malic acid 100 g^−1^ FW)		(mg NH _4_ ^+^ kg^−1^ FW)	(μg kg^−1^ FW)
No application	No application	62.4 e	2.91 g	65.25 h	38.08 a	162.83 e	4.64 a	3.79 d	0.38 d	0.41 c
	20%	70.4 c	3.08 f	67.58 g	34.75 b	180.08 d	4.35 b	3.85 cd	0.17 f	0.20 e
	30%	68.5 cd	3.12 f	71.75 e	27.91 de	184.25 d	4.15 c	3.86 cd	0.20 ef	0.24 de
1 mM	No application	64.08 e	3.25 e	69.66 f	30.91 c	170.50 de	4.03 d	3.47 e	0.18 ef	0.24 de
	20%	65.8 de	3.52 d	79.75 d	29.41 cd	199.75 c	3.69 f	3.74 d	0.25 e	0.27 d
	30%	78.3 b	3.82 b	84.00 b	26.50 e	199.16 c	3.69 f	3.96 c	0.40 d	0.40 c
2 mM	No application	71.5 c	3.69 c	81.66 c	27.50 e	176.50 de	3.81 e	3.76 d	0.49 c	0.43 c
	20%	81.08 b	3.88 b	85.16 ab	26.58 e	254.66 b	3.58 g	4.14 b	0.56 b	0.55 b
	30%	98.6 a	4.14 a	86.58 a	23.00 f	313.83 a	3.35 h	4.30 a	0.68 a	0.69 a

*Note:* Means within each column followed by the same letter are not significantly different by least significant difference test (*p* ≤ 0.05).

### 3.2 Fruit Weight Loss

The main effects of SA, AV gel, and CE significantly influenced fruit weight loss (Table 1). Compared with untreated fruit, fruit weight loss decreased by 14.27% and 48.55% under 1 mM and 2 mM SA applications, respectively (Table 3). Increasing AV gel concentration led to a progressive reduction in fruit weight loss (Table 3), with the highest and lowest values recorded in untreated fruit (14.72%) and fruit treated with 30% AV gel (10.55%), respectively. Mean comparisons (Table 4) showed that fruit weight loss decreased significantly following application of 150 and 300 ppm CE compared with no application, with average reductions of 21.12% and 28.32%, respectively.

**TABLE 3:** Mean comparison of the main effect of salicylic acid and *aloe vera* gel application on some studied traits of peach ( *Prunus persica* L.).

	Fruit weight loss	Vitamin C	Total antioxidant capacity
	(%)	(mg 100 g^−1^ FW)	(μmol TE g^−1^ FW)
Salicylic acid			
No application	15.55 a	14.91 c	351.83 c
1 mM	13.33 b	24.13 b	413.75 b
2 mM	8.00 c	32.02 a	478.02 a
*Aloe vera* gel			
No application	14.72 a	19.44 c	383.11 c
20%	11.61 b	23.58 b	413.55 b
30%	10.55 c	28.05 a	446.94 a

*Note:* Means within each column followed by the same letter are not significantly different by least significant difference test (*p* ≤ 0.05).

**TABLE 4:** Mean comparison of the main effect of clove extract application on some studied traits of peach ( *Prunus persica* L.).

Clove extract	Fruit weight loss	Fruit texture firmness	Total phenolic content	Vitamin C	Total antioxidant capacity	Total acidity	Ammonium content	Toluene
	(%)	(kg cm^−2^)	(mg GAE 100 g^−1^ FW)	(mg 100 g^−1^ FW)	(μmol TE g^−1^ FW)	(g malic acid 100 g^−1^ FW)	(mg NH _4_ ^+^ kg^−1^ FW)	(μg kg^−1^ FW)
No application	14.72 a	3.28 c	187.94 c	19.44 c	383.11 c	4.16 a	0.85 c	0.36 b
150 ppm	11.61 b	3.49 b	206.02 b	23.58 b	413.55 b	3.87 b	0.96 b	0.34 b
300 ppm	10.55 c	3.69 a	219.88 a	28.05 a	446.94 a	3.74 c	1.01 a	0.44 a

*Note:* Means within each column followed by the same letter are not significantly different by least significant difference test (*p* ≤ 0.05).

### 3.3 Fruit Texture Firmness

Averaged across SA treatments, application of 20% and 30% AV gel resulted in higher fruit texture firmness, corresponding to increases of approximately 5.84% and 11.05%, respectively, compared with no AV application (Table 2). Fruit treated with CE exhibited higher firmness than untreated fruit, with increases of 5.42% and 11.11% observed at 150 and 300 ppm, respectively (Table 4).

### 3.4 Fruit Flesh

**FIGURE 2:**
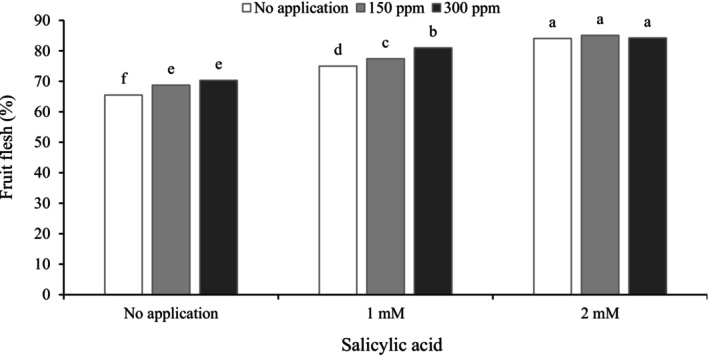
Mean comparison of interaction effect of salicylic acid × clove extract on fruit flesh of peach ( *Prunus persica* L.). Means followed by the same letter are not significantly different by least significant difference test (*p* ≤ 0.05).

Regardless of AV gel application, fruit flesh percentage increased significantly in response to SA treatments (Table 2). The highest flesh percentages were recorded in fruit treated with 2 mM SA combined with 30% AV gel (86.58%) and 20% AV gel (85.16%), whereas the lowest value (65.25%) was observed in fruit receiving neither SA nor AV gel (Table 2). Similarly, the highest and lowest flesh percentages were observed in fruit treated with 2 mM SA × 150 ppm CE (85.08%) and in untreated fruit (65.50%), respectively (Figure 2). At 2 mM SA, all CE levels resulted in statistically similar flesh percentages. Regardless of SA treatment, increasing CE concentration increased fruit flesh percentage.

### 3.5 Decay Percentage

Among treatments, the highest decay percentage (38.08%) was observed in fruit receiving neither SA nor AV gel (Table 2). At each AV gel level, increasing SA concentration reduced decay percentage. Averaged across SA treatments, application of 20% and 30% AV gel reduced decay percentage by approximately 5.35% and 19.0%, respectively, compared with untreated fruit (Table 2).

### 3.6 Total Phenolic Content

Regardless of AV gel application, total phenolic content increased with increasing SA concentration. The highest phenolic content (313.83 mg GAE 100 g^−1^ FW) was observed in fruit treated with 2 mM SA and 30% AV gel (Table 2). At each SA level, increasing AV gel concentration resulted in higher phenolic content. However, no significant differences were observed among AV gel treatments at the same SA level, and application of 20% and 30% AV gel at 1 mM SA resulted in similar phenolic contents.

### 3.7 Vitamin C

The main effects of SA, AV gel, and CE significantly affected vitamin C content (Table 1). Vitamin C increased by 38.2% and 53.4% under 1 mM and 2 mM SA treatments, respectively, compared with untreated fruit (Table 3). Mean comparisons showed that vitamin C content increased significantly by 17.5% and 32.2% following application of 20% and 30% AV gel, respectively, compared with no application (Table 3). Increasing CE concentration also resulted in higher vitamin C content, with the highest and lowest values observed at 300 ppm (28.05 mg 100 g^−1^ FW) and no CE application (19.44 mg 100 g^−1^ FW), respectively.

### 3.8 Total Antioxidant Capacity

Analysis of variance indicated that SA, AV gel, and CE significantly affected total antioxidant capacity (Table 1). Compared with untreated fruit, total antioxidant capacity increased by 14.9% and 26.3% under 1 mM and 2 mM SA treatments, respectively (Table 3). Application of 20% and 30% AV gel increased total antioxidant capacity by an average of 7.36% and 14.28%, respectively, compared with no application (Table 3). Fruit treated with 300 ppm CE exhibited the highest total antioxidant capacity (446.94 μmol TE g^−1^ FW), whereas untreated fruit showed the lowest value (383.11 μmol TE g^−1^ FW), representing a difference of 14.28% (Table 4).

### 3.9 Total Acidity

Analysis of variance showed that the main effect of CE and the interaction between SA and AV gel significantly affected total acidity at the 1% probability level (Table 1). At all SA levels, application of 20% and 30% AV gel increased total acidity compared with no AV gel application (Table 2). The highest total acidity (4.64 g malic acid 100 g^−1^ FW) was observed in fruit receiving neither SA nor AV gel, whereas the lowest value (3.35 g malic acid 100 g^−1^ FW) occurred in fruit treated with 2 mM SA without AV gel (Table 2). Regarding CE treatments, the highest total acidity was recorded in untreated fruit (4.16 g malic acid 100 g^−1^ FW), which was 6.97% and 10.09% higher than values recorded at 150 and 300 ppm CE, respectively.

### 3.10 Juice pH

Analysis of variance showed that the interaction between SA and AV gel significantly affected juice pH (Table 1). The highest juice pH value (4.30) was observed in fruit treated with 2 mM SA and 30% AV gel (Table 2). The lowest juice pH (3.47) was recorded in fruit treated with 1 mM SA without AV gel, which was even lower than that of untreated fruit. At the no‐SA level, all AV gel concentrations resulted in similar juice pH values.

### 3.11 Ammonium Content

At 1 mM and 2 mM SA levels, ammonium content increased with increasing AV gel concentration (Table 2). Averaged across SA treatments, no significant difference was observed between no AV gel application and 20% AV gel application. The highest ammonium content (0.68 mg kg^−1^ FW) was observed in fruit treated with 2 mM SA and 30% AV gel, whereas the lowest value (0.17 mg kg^−1^ FW) was recorded in fruit without SA application (Table 2). At the no‐SA level, ammonium content decreased with increasing AV gel concentration. Regarding CE treatments, the highest ammonium content was observed at 300 ppm CE (1.01 mg kg^−1^ FW), which was 15.8% and 4.9% higher than values recorded in untreated fruit and fruit treated with 150 ppm CE, respectively (Table 4).

### 3.12 Toluene

Under no‐SA conditions, application of 20% and 30% AV gel reduced toluene content compared with no AV gel application (Table 2). Averaged across AV gel levels, fruit treated with SA (1 and 2 mM) exhibited higher toluene content than untreated fruit (Table 2). The highest toluene content (0.69 μg kg^−1^ FW) was observed in fruit treated with 2 mM SA and 30% AV gel, followed by fruit treated with 2 mM SA and 20% AV gel (0.55 μg kg^−1^ FW). These values represented increases of 37.6% and 21.8%, respectively, compared with untreated fruit (Table 2). Regarding CE treatments, the lowest toluene content (0.36 μg kg^−1^ FW) was observed in fruit without CE application (Table 4). Increasing CE concentration increased toluene content, with the highest value recorded at 300 ppm CE. However, fruit treated with 150 ppm and no CE did not differ significantly.

## 4 Discussion

The present study investigated the effects of SA, AV gel coating, and CE, individually and in combination, on postharvest quality attributes of peach. Fruit weight represents an integrated outcome of growth processes prior to harvest and metabolic stability during storage. In the present investigation, fruit weight responded markedly to SA, AV gel, and CE applications, indicating that these treatments influenced not only surface‐level preservation but also internal physiological regulation. Fruits treated with higher SA concentrations, particularly 2 mM, consistently exhibited superior weight retention compared with untreated controls. This response suggests that SA contributed to sustained cellular integrity and delayed senescence rather than merely reducing superficial water loss (Keshavarz et al. 2016, 2024). SA is known to regulate stomatal behavior and suppress ethylene biosynthesis (Keshavarz et al. 2016, 2021). By moderating ethylene‐related processes and slowing catabolic metabolism, SA‐treated fruits maintained tissue density and cellular cohesion for a longer period. The enhanced fruit weight observed under SA treatments may therefore be attributed to reduced catabolic activity and improved maintenance of cellular turgor, which led to improved weight retention during storage. Reduction in weight loss in SA‐treated peaches is consistent with findings in other climacteric fruits where SA inhibited respiration and transpiration processes (Keshavarz et al. 2018; Morsalpour and Rastegar 2025). Similar outcomes have been documented in grapes (*Vitis vinifera L*.), where chitosan combined with AV coating reduced the incidence of decay and weight loss relative to untreated fruits (Mousavi and Abbaszadeh 2022). AV gel, functioning as a semi‐permeable coating, further limits gas exchange and water vapor diffusion, creating a modified internal atmosphere that slows metabolic degradation (Saleem et al. 2022). Similarly, CE contains phenylpropanoids such as eugenol, which exert antimicrobial and antioxidant effects; therefore, it limited tissue degradation and secondary metabolic losses and indirectly preserved tissue integrity and water retention (Baran Zehi et al. 2020; Keshavarz and Sadegh‐Ghol‐Moghadam 2017). The strong inverse association observed between fruit weight and fruit weight loss across treatments reinforces this interpretation (*r* = −0.69**), indicating that weight retention primarily reflected reduced transpirational and respiratory losses. AV gel further enhanced fruit weight preservation, with 30% gel outperforming the 20% concentration. The positive correlation between fruit weight and firmness (*r* = 0.74**) supports the view that structural preservation played a decisive role in mass retention. Collectively, these findings indicate that fruit weight preservation emerged from the combined effects of reduced water loss, delayed senescence, and improved tissue stability rather than from a single isolated mechanism.

Fruit weight loss is a sensitive indicator of postharvest stress, reflecting cumulative water loss, respiratory activity, and structural integrity of fruit tissues during storage (Koyuncu et al. 2019). In this study, all treatments significantly reduced weight loss compared with the untreated control, with the greatest reductions observed under higher levels of SA treatment. SA at 2 mM was particularly effective, suggesting a strong influence on metabolic regulation by modulating membrane stability and suppressing ethylene‐mediated respiration (Sinha et al. 2021). The reduction in weight loss under SA treatment is likely linked to suppressed respiration and slower progression of senescence‐related processes. This interpretation is supported by the consistent negative relationship between fruit weight loss and firmness, indicating that fruits experiencing less moisture loss also retained stronger cell wall integrity. AV gel treatments further reduced weight loss in a concentration‐dependent manner, with 30% gel providing the greatest protection, acts as moisture barriers, reducing transpiration by sealing surface microcracks and limiting vapor pressure gradients between fruit tissue and the surrounding environment (Saleem et al. 2022). This coating likely limited water vapor diffusion while simultaneously creating a modified internal atmosphere that slowed oxidative metabolism. CE treatments followed a similar trend, with higher concentrations producing lower weight loss and further contributing by reducing microbial colonization, which otherwise accelerates tissue breakdown and water loss. The pronounced reduction in fruit weight loss under combined treatments suggests a synergistic interaction among biochemical, physical, and antimicrobial mechanisms. The association between reduced weight loss and increased phenolic and antioxidant levels (*r* = −0.71**, *r* = −0.86**, respectively) suggests that enhanced oxidative stability contributed to membrane preservation, thereby limiting water escape. Overall, the convergence of physical protection and biochemical stabilization explains the substantial reductions in fruit weight loss observed across treatments.

Firmness is a critical sensory and commercial attribute, reflecting the integrity of cell walls degradation and middle lamella structures. In the present study, firmness was significantly improved by all treatments, with maximum values recorded under 2 mM SA, 30% AV gel, and 300 ppm CE. These responses indicate delayed softening and reduced enzymatic degradation of cell wall polysaccharides. SA‐treated fruits showed consistently higher firmness, suggesting inhibition of enzymes involved in pectin solubilization and cell wall–degrading enzymes such as polygalacturonase and cellulose, thereby preserving tissue rigidity (Karami et al. 2016). AV gel also contributed to firmness preservation, likely by reducing dehydration stress, oxygen diffusion, limiting oxidative reactions, and slowing metabolic activity associated with cell wall disassembly. Additionally, phenolic compounds in CE may inhibit pectinolytic enzymes which may be attributed to reduced microbial activity and oxidative damage at the tissue level therefore, strengthen cell wall structures through cross‐linking interactions. Similar firmness‐preserving effects have been reported in coated and SA‐treated fruits, supporting the mechanistic interpretation of delayed cell wall disassembly (Taher et al. 2022). The inverse relationship between firmness and decay percentage (*r* = −0.72**) observed across treatments indicates that firmer fruits were less vulnerable to pathogen invasion and suggests that decay control was closely linked to the maintenance of both physical structure and internal biochemical defenses. These findings highlight firmness as a pivotal trait linking physical, biochemical, and pathological aspects of postharvest performance. The positive association between firmness and fruit weight (*r* = 0.74**) further emphasizes the role of structural integrity in maintaining overall fruit quality.

Fruit flesh percentage reflects the proportion of edible tissue, while decay represents one of the most critical sources of postharvest loss. Treatments in the present study significantly increased flesh percentage compared with untreated fruits, particularly under higher SA levels combined with AV gel or CE. The enhanced flesh percentage can be attributed to reduced dehydration and limited tissue collapse during storage. SA stabilizes membrane structures and reduces electrolyte leakage, thereby maintaining cellular volume (Jahani et al. 2021). AV gel coatings further reduce moisture loss, preserving flesh mass relative to total fruit weight. The antimicrobial properties of CE likely reduce localized tissue breakdown caused by microbial invasion and further reinforce resistance to decay‐causing organisms. Fruit weight showed a positive correlation with firmness (*r* = 0.74**), phenolic content (*r* = 0.81**), and fruit flesh percentage (*r* = 0.60**), indicating that treatments promoting biochemical stability also promote higher fruit mass retention with delayed senescence and preserved tissue integrity. This relationship suggests that reduced water loss and metabolic degradation directly contributed to the maintenance of fruit structure. Firmness and flesh percentage themselves were very strongly correlated (*r* = 0.88**), highlighting the close dependence of texture preservation on maintenance of parenchymal tissue. These correlations support the observed effects of higher SA concentrations and AV gel coatings, which limited transpiration and respiration, thereby stabilizing cellular organization and reducing softening. Similar relationships between firmness and flesh integrity have been reported in peaches and nectarines subjected to postharvest coatings and antioxidant treatments (Nasrin et al. 2025).

The marked reduction in decay under treated conditions indicates effective suppression of microbial development and delayed tissue senescence. SA likely enhanced endogenous defense responses and induces systemic resistance by activating defense‐related enzymes and phenylpropanoid metabolism, enhancing the fruit's innate resistance to pathogens (Yang et al. 2020) while AV gel provides a physical barrier that limits pathogen entry, and its inherent bioactive compounds exhibit antifungal activity. In addition, the lower decay incidence in AV‐treated fruits likely results from reduced oxygen availability at the fruit surface and inhibition of microbial growth, a mechanism that has been reported for other stone fruits and may have practical implications for reducing postharvest losses without reliance on synthetic fungicides (Hasan et al. 2021). CE, rich in antimicrobial constituents and eugenol, directly inhibits fungal growth and disrupts microbial cell membranes and further reinforces resistance to decay‐causing organisms (Abdul‐Aziz et al. 2023). The strong decay suppression observed under combined treatments highlights the advantage of integrating induced resistance, physical exclusion, and direct antimicrobial action. This multi‐layered defense strategy is particularly effective in reducing postharvest losses due to decay. Additionally, fruit weight was negatively correlated with decay (*r* = −0.57**), suggesting that dehydration and tissue collapse created favorable conditions for pathogen invasion. These relationships reinforce the conclusion that edible coatings and SA delay decay primarily by preserving physical barriers and membrane integrity, rather than solely through direct antimicrobial action (Zhao et al. 2021). AV gel likely contributed to this effect by forming a semi‐permeable coating that reduced moisture loss and surface cracking.

Total phenolic content is widely recognized as an indicator of antioxidant potential and stress responsiveness, play a central role in antioxidant defense and pathogen resistance, and their accumulation is often associated with enhanced postharvest quality (Keshavarz Mirzamohammadi, Modarres‐Sanavy, et al. 2021). In this study, phenolic levels increased significantly under all treatments, with the highest concentrations recorded in fruits receiving 2 mM SA and 300 ppm CE. This enhancement suggests activation of secondary metabolic pathways associated with defense and stress mitigation and likely reflects both reduced catabolism and enhanced synthesis via defense‐related pathways activated by SA and antioxidative extracts like CE. SA is known to stimulate phenylpropanoid metabolism, which might be contributed to the elevated phenolic accumulation observed (Jahani et al. 2021). SA likely activates phenylpropanoid metabolism and the phenylalanine ammonia‐lyase pathway, leading to greater accumulation of phenolic compounds and improved free radical scavenging capacity. This mechanistic route has been reported in mango fruits treated with SA and linked to delayed senescence by mitigating oxidative stress (Morsalpour and Rastegar 2025). AV gel treatments also supported higher phenolic retention, potentially by limiting oxidative degradation during storage while CE can contribute exogenous phenolic or induce endogenous synthesis through stress signaling. CE may have contributed both directly, through its own phenolic constituents, and indirectly, by inducing endogenous synthesis (El Rayess et al. 2023). Total Phenols content were negatively correlated with decay (*r* = −0.54**), supporting the dual role of phenolic compounds in both antioxidant defense and antimicrobial resistance. This dual contribution likely explains the pronounced phenolic enhancement observed under CE treatments. Moreover, CE is rich in eugenol and other phenolic constituents with strong antioxidant and antimicrobial properties (Abdul‐Aziz et al. 2023). Natural extracts from clove have been reported to inhibit microbial spoilage and enhance the antioxidant status of treated produce (El Rayess et al. 2023). Although the literature on CE specifically in peaches remains limited, studies with other spice extracts and essential oils indicate a capacity to suppress fungal growth and delay senescence when used as postharvest treatments (Álvarez‐García et al. 2023). The presence of antimicrobial agents in CE can also diminish decay incidence by inhibiting pathogen colonization, as supported by the antimicrobial properties of phenolic extracts in postharvest systems. The strong positive correlation between total phenolic content and total antioxidant activity (*r* = 0.71**) confirms the central contribution of phenolic compounds to the fruit's antioxidant system.

Vitamin C is a nutritionally important compound that is highly susceptible to oxidative loss during postharvest storage. The improved retention of vitamin C under SA treatment suggests reduced oxidative degradation, possibly due to enhanced antioxidant enzyme activity and suppressed oxidative stress. AV gel coatings likely limited oxygen diffusion, thereby slowing ascorbic acid oxidation. CE treatments further supported vitamin C retention, consistent with their antioxidant properties. The positive association between vitamin C content and total antioxidant activity (*r* = 0.89**) indicates that preservation of this compound contributed meaningfully to overall antioxidant capacity. Vitamin C showed negative relationships with fruit weight loss (*r* = −0.81**) and decay (*r* = −0.74**), indicating that fruits experiencing lower physiological stress maintained higher nutritional quality throughout storage. Treatments that reduce oxidative stress—such as SA, AV, and CE—may slow vitamin C loss by attenuating reactive oxygen species and stabilizing cellular antioxidants. This mechanism mirrors findings in coated fruits where ascorbic acid levels remained higher with AV and natural extracts (Cao et al. 2025; Xiao et al. 2011). The improved retention of vitamin C under combined treatments underscores the importance of oxidative stress management in postharvest quality preservation and highlights the nutritional benefits of these interventions.

Total antioxidant activity represents the cumulative effect of multiple antioxidant constituents within the fruit and followed trends similar to phenolic and vitamin C content, increasing significantly in treated fruits. SA induces antioxidant enzyme activity, including catalase and peroxidase, while also promoting phenolic biosynthesis. AV gel coatings reduce oxidative load by modifying the internal atmosphere. CE provides additional antioxidant compounds, contributing directly to measured antioxidant capacity. The elevated antioxidant capacity under combined treatments suggests improved resistance to oxidative damage, which is critical for maintaining fruit quality during extended storage (Keshavarz Mirzamohammadi, Tohidi‐Moghadam, and Hosseini 2021). Strong positive relationships were observed between antioxidant activity, total phenolic content and vitamin C levels, highlighting the integrated nature of the fruit's antioxidant system. Antioxidant activity was inversely related to decay incidence and weight loss, suggesting that enhanced oxidative defense played a critical role in maintaining both physical integrity and shelf life. These findings indicate that treatments promoting antioxidant accumulation also indirectly supported other quality traits by mitigating oxidative damage and delaying senescence‐associated deterioration.

Storage‐related declines in total acidity and shifts in juice pH are well‐documented in fruit physiology and generally result from organic acid catabolism during respiration (Ezeike et al. 2022). The retention of higher acidity under treated conditions indicates delayed metabolic degradation and improved flavor stability. Total acidity declined during storage but was better maintained in treated fruits, particularly under AV gel and SA applications. Organic acids are substrates for respiration, and their depletion is indicative of advanced senescence (Keshavarz 2020). SA reduces respiration rate, slowing the consumption of organic acids. AV gel coatings further limit metabolic activity by restricting oxygen availability. CE also contributed to acidity retention, likely by moderating respiration rates and slowing organic acid degradation. Total acidity showed negative correlations with firmness (*r* = −0.88**) and flesh percentage (*r* = −0.86**), as well as a positive correlation with decay (*r* = 0.69**) reflect the metabolic consumption of organic acids during advanced ripening and senescence and suggesting that metabolic stability was closely linked to structural and biochemical preservation. Maintaining stable juice pH is essential for sensory quality and microbial stability (Yang et al. 2020; Hosseini et al. 2023b). As acidity declined, tissue breakdown accelerated, resulting in higher decay incidence. The stabilization of pH under SA and AV gel treatments reflects reduced organic acid catabolism and controlled metabolic activity. Therefore, the observed pH moderation supports the effectiveness of the applied treatments in delaying ripening‐associated biochemical changes. Total acidity was inversely related to juice pH (*r* = −0.41**), increasing more slowly in treated fruits further confirms the tight regulation of organic acid metabolism during postharvest storage. Treatments that suppress respiration, such as SA and AV coatings, help conserve organic acids and maintain lower pH longer into storage, which can contribute to better taste and reduced microbial spoilage. Juice pH was positively correlated with fruit weight (*r* = 0.66**) and total phenolic content (*r* = 0.81**), indicating that treatments maintaining higher physiological activity and biochemical stability also preserved juice characteristics. Research in grapes, for example, showed that SA and AV maintained higher titratable acidity compared to controls (Mousavi and Abbaszadeh 2022; Hosseini et al. 2023a).

Ammonium content and toluene accumulation provide insight into nitrogen metabolism and volatile compound dynamics during storage and these compounds are associated with protein degradation and senescence‐related metabolic imbalance (Aydin et al. 2025; Zhang et al. 2020). In this study, ammonium content increased with storage duration but was significantly lower in treated fruits which may reflect altered nitrogen assimilation patterns and reduced protein breakdown. SA helps maintain nitrogen metabolism balance by reducing proteolysis and enhancing stress tolerance. AV gel coatings limit metabolic stress, while CE reduces microbial‐induced protein degradation (Mohammed et al. 2018; Crutcher et al. 2022). Lower ammonium levels under combined treatments indicate delayed senescence and improved metabolic stability. Toluene production is often associated with anaerobic metabolism and tissue stress. Treated fruits exhibited significantly lower toluene levels, particularly under optimized combinations of SA and AV coatings. However, it showed a positive association with ammonium content (*r* = 0.91**), confirming that both compounds act as senescence‐related metabolic indicators and coordinated metabolic adjustments under stress‐modulating treatments. By moderating respiration and preventing excessive oxygen depletion, AV gel coatings and SA treatments help avoid the shift toward anaerobic pathways that generate volatile stress markers such as toluene. Reduced toluene accumulation thus reflects improved internal atmospheric balance and reduced physiological stress. Notably, both parameters exhibited negative relationships with decay percentage (*r* = −0.47** and *r* = −0.44** ammonium and toluene, respectively), suggesting that their accumulation did not signal quality deterioration but rather a modified metabolic state associated with enhanced stress tolerance. Also, this supports the idea that lower ammonium/toluene levels reflect delayed protein degradation and reduced metabolic stress. Their positive association with fruit weight (*r* = 0.68** and *r* = 0.69** ammonium and toluene, respectively) and total phenolic content (*r* = 0.67** and *r* = 0.73** ammonium and toluene, respectively) suggests that treatments preserving overall metabolic integrity also suppressed undesirable nitrogenous and volatile by‐products.

## 5 Conclusions

Postharvest senescence in climacteric fruits like peach is driven by increased respiration, ethylene production, cell wall breakdown, and oxidative stress. Treatments like SA and antioxidant‐rich extracts can modulate these processes by (1) suppressing ethylene biosynthesis and action, thereby delaying ripening and softening; (2) enhancing antioxidant enzyme activities that mitigate ROS accumulation; and (3) maintaining membrane integrity, reducing electrolyte leakage and associated quality loss. These mechanisms are widely recognized in postharvest biology and underpin many recent treatment strategies implementing phytohormones and natural coatings. Edible coatings can create a semi‐permeable barrier around the fruit surface that reduces gas exchange, slows respiration, and limits moisture loss, thereby extending shelf life. Total acidity and juice pH are key indicators of metabolic status and sensory quality. In this study, treated fruits generally maintained higher acidity and lower pH values compared with the control, reflecting delayed ripening and reduced utilization of organic acids during storage. The synergistic effects observed under combined treatments highlight the complementary mechanisms of hormonal regulation (SA), physical barrier formation (AV gel), and biochemical protection (CE), which represent a promising, eco‐friendly alternative to conventional postharvest chemical preservatives. These treatments are generally regarded as safe (GRAS) and compatible with organic production standards. These findings indicate that integrated postharvest treatments not only preserve overall fruit mass but also enhance the proportion of marketable, edible tissue, which is of significant commercial importance. Their application can reduce reliance on synthetic fungicides and retard physiological deterioration under moderate storage conditions, potentially extending shelf life while preserving nutritional and sensory quality—a priority in commercial stone fruit supply chains. While the overall trends are clear, future studies could refine optimal concentrations for commercial application and explore the molecular regulation of quality traits, such as gene expression of cell wall enzymes and antioxidant systems. Additionally, sensory analysis and consumer acceptance studies would further validate the practical utility of these treatments.

## Funding

The authors have nothing to report.

## Ethics Statement

All plant specimens were collected with permissions.

## Conflicts of Interest

The authors declare no conflicts of interest.

## Data Availability

All data supporting the findings of this study are included within the article.
